# Errorless and errorful learning modulated by transcranial direct current stimulation

**DOI:** 10.1186/1471-2202-12-72

**Published:** 2011-07-22

**Authors:** Anke Hammer, Bahram Mohammadi, Marlen Schmicker, Sina Saliger, Thomas F Münte

**Affiliations:** 1 Dept. of Neurology, University of Lübeck, Ratzeburger Allee 160, 23538 Lübeck, Germany; 2 Neurology, International Neuroscience Institute (INI), Rudolf-Pichlmayr-Straße 4, 30625, Hannover, Germany; 3 Institute of Medical Psychology, Otto-von-Guericke University, Leipziger Straße 44, 39120 Magdeburg, Germany

## Abstract

**Background:**

Errorless learning is advantageous over trial and error learning (errorful learning) as errors are avoided during learning resulting in increased memory performance. Errorful learning challenges the executive control system of memory processes as the erroneous items compete with the correct items during retrieval. The left dorsolateral prefrontal cortex (DLPFC) is a core region involved in this executive control system. Transcranial direct current stimulation (tDCS) can modify the excitability of underlying brain functioning.

**Results:**

In a single blinded tDCS study one group of young healthy participants received anodal and another group cathodal tDCS of the left DLPFC each compared to sham stimulation. Participants had to learn words in an errorless and an errorful manner using a word stem completion paradigm. The results showed that errorless compared to errorful learning had a profound effect on the memory performance in terms of quality. Anodal stimulation of the left DLPFC did not modulate the memory performance following errorless or errorful learning. By contrast, cathodal stimulation hampered memory performance after errorful learning compared to sham, whereas there was no modulation after errorless learning.

**Conclusions:**

Concluding, the study further supports the advantages of errorless learning over errorful learning. Moreover, cathodal stimulation of the left DLPFC hampered memory performance following the conflict-inducing errorful learning as compared to no modulation after errorless learning emphasizing the importance of the left DLPFC in executive control of memory.

## Background

The executive control system enables us to learn, to plan behavior, and to inhibit incorrect behavior. With regard to memory functions, often several pieces of associatively linked information (e.g. several names associated to one face) may be available during retrieval and thus incorrect information might interfere with the correct information. Clinically, the inability to distinguish between correctly and incorrectly associated information might lead to neuropsychological conditions such as frontal confabulatory disorder [1]. In experimental work on memory-failures, one important theory to explain false memories, for example the Deese-Roediger-McDermott paradigm [2,3], draws on the notion of falsely activated information [4]. In other words, there appears to be a need to control for and reject falsely associated information during memory retrieval.

One method to study the role of executive control processes during memory retrieval is to modulate the excitability of the prefrontal cortex (PFC). The PFC plays an essential role in the integration of information and the management of multiple tasks [5] as it is crucial in conducing higher cognitive functions, i.e. executive functions including working memory, planning, goal-oriented behaviour, role learning, attention and inhibition and control [e.g. 6, 7-23]. Another method is the use of different encoding modes, i.e. errorful and errorless learning, designed to manipulate the presence of interfering information, which needs to be controlled during memory retrieval [24-26]. Hence, the combination of both, the modulation of the excitability of the PFC and the use of different encoding modes enables us to investigate the importance of executive control processes during memory retrieval depending on the PFC.

As mentioned above, the modulation of the excitability of a specific region may help to clarify the role of this region during a cognitive task. Transcranial direct current stimulation (tDCS) is a noninvasive technique for such a modulation as it changes the cortical excitability depending on the polarity of the applied weak current. In general, anodal tDCS increases and cathodal tDCS decreases the neural firing rates, probably due to an induced change in the resting membrane potential [27-29]. These changes lead to corresponding changes in cortical functions: e.g. the excitation of the motor cortex by anodal tDCS leads to increased motor responses and the inhibition by cathodal tDCS leads to decreased motor responses [29] resulting in improved or in inhibited functioning of the motor cortical areas. In relation to more complex functions the same has been found to be true for memory processing [30-34], executive functions such as verbal fluency [35], language processing [36] or decision making [37] in healthy subjects [for a review see 38]. Similar findings have been reported after transcranial magnetic stimulation (TMS), another method to modulate the excitability of the underlying cortical tissue [e.g. 39, 40-43].

With regard to memory processes, the effects of tDCS have been mainly assessed with regard to working memory (WM). Anodal but not cathodal stimulation (1 mA, 10 min, 35-cm^2^-sized electrodes) over the left prefrontal cortex resulted in improved performance accuracy on a visual letter WM task [31]. However, Marshall et al. [32] reported slowed reaction time in a visual letter WM task during both anodal and cathodal bilateral stimulation over the DLPFC, suggesting that any kind of electrical stimulation hampers neuronal processes related to response selection and preparation. Ohn et al. [34] showed a time dependent effect of anodal stimulation over the left PFC (1 mA, 35 cm^2^-sized electrodes) on WM reporting an increased number of correct responses after 30 minutes of stimulation compared to sham, but earlier measurements or error rates did not reveal any stimulation effects. Boggio et al. [44] reported that continuous tDCS for 20 min at 2 mA (but not at 1 mA) using the same-sized electrodes improved WM in patients with Parkinson's disease.

Other studies focused on different memory components. Marshall and colleagues [33] investigated consolidation of declarative memories and found that bilateral anodal direct current stimulation at the DLPFC affected declarative memory when applied during sleep. Boggio et al. [45] found that false memories were reduced by 73% when anodal tDCS is applied to the anterior temporal lobes throughout the encoding and retrieval stages. However, veridical memories remained unchanged after stimulation. Finally, Elmer et al. [46] investigated tDCS effects on short-term learning and subsequent long-term retrieval of auditorily presented verbal material, i.e. wordlist learning with immediate and delayed recall. Cathodal stimulation but not anodal stimulation of the left prefrontal cortex (1.5 mA) disrupted short-term verbal learning but did not hamper longer lasting consolidation processes that are mainly known to be related to mesial temporal lobe areas. The stimulation of the right prefrontal cortex failed to modulate verbal short-term learning and subsequent long-term retrieval. Further research has evidenced facilitation of learning and memory processes by tDCS application to the PFC [27,47].

In summary one can conclude that tDCS is a non-invasive tool to modulate cognitive processes. However, polarity, intensity, duration and site of stimulation, as well as the size of the electrodes are important parameters in the effects of tDCS on memory processes that are not entirely understood [38].

On the cognitive side, one method to study the role of executive control processes during memory retrieval is the use of different encoding modes, errorful and errorless learning [24,26,48]. Errorful learning resembles the typical trial-and-error approach. During learning a number of errors are introduced until the correct response is produced. Exactly these errors are likely to cause interference and false memories/intrusions at retrieval. In contrast, during errorless learning - a managed learning mode - only the correct information is introduced and errors are avoided during the learning process reducing the later interference during recall. Errorless learning has been found to lead to profound enhancement of memory retrieval in particular in neuropsychiatric conditions associated to memory deficits such as brain injury [24,49-51], Alzheimer disease [52-54] and schizophrenia [55-58]. A critical review of this literature has been provided by Clare and Jones [48]. Following errorful learning, memory impaired patients may not be able to use the remaining resources of their implicit memory, because they are not able to differentiate between errors made during learning and the correctly learned information [24]. Consequently, such patients benefit from errorless learning compared to errorful learning [24,49-53,55-58], as errors are avoided during the studying phase. Baddeley and Wilson [24] assume that the disadvantage for errorful learning in a word fragment completion task is based on the increased activation level of wrong words of the learning phase which leads to interference. In contrast, this interference is diminished within the errorless modus as only one stimulus was presented during learning. In cases, in which retrieval is based mostly on implicit memory processes, errors are committed because it is not possible to differentiate between concurrent items. Thus, episodic or explicit memory processes are needed to resolve the interference in the errorful modus. This interpretation is supported by electrophysiological studies [25,26,59] focusing on the so called error related negativity (ERN). Contrasting errorful and errorless learning implemented in a word-stem completion task resulted in a learning mode effect in particular for the ERN [25,26,59], which was thought to reflect aspects related to the memory decision. The modulation of the ERN amplitude in relation to memory decisions was interpreted as reflecting the activity of an internal monitoring device assessing the activation of the two possible decisions, i.e. the veridicality of retrieved memory traces [25,26,59] or, as an alternative interpretation, variations of the ERN amplitude in errorless and errorful learning might be partially explained by the subjects' perceived likelihood of making an error [25,59]. For both, (decision about the veridicality of retrieved memory traces or the error prevention in different levels of perceived error likelihood) executive control mechanisms of retrieved memory are indispensable.

The ERN is a response-related potential, whose neural source has been consistently found in the posterior medial frontal cortex as shown by brain potential source localization studies [23,60-62] and error-related fMRI activity [63-65] with additional contributions from the lateral prefrontal cortex [7,63,66-69]. The PFC, the target region of the present investigation, is known to be involved in higher executive functions as already described above. It is still unclear whether the left PFC is clearly associated to memory retrieval processes or executive processes such as monitoring as the left PFC was activated for both [18]. Elmer and colleagues [46] modulated the PFC bilaterally based on the HERA hemispheric encoding/retrieval model (HERA) [70] as it states that the left PFC is more involved in episodic memory encoding and the right PFC is more involved in episodic memory retrieval. However, the findings of Elmer et al. suggested a lateralization as a function of the material (i.e. verbal material involving the left language dominant hemisphere) rather than the stage (i.e., encoding or retrieval). As we focus on a verbal task, the targeted region in the present investigation was the left PFC. In order to modulate the activity of the left PFC we used tDCS to examine its role in executive control of memory processes using the two different learning modes, i.e. errorless and errorful learning. The combination of both, changed excitability of left prefrontal regions and the modulation of conflict during memory performance aids us to clarify the specific role of the left PFC of the memory system during executive control of memory processes.

Only a paucity of the tDCS studies to date has explored the modulation of prefrontal areas during explicit memory tasks and to our knowledge, none of the studies modulated conflicting information during encoding. We hypothesised in view of the findings of some neuroimaging studies [63-65] and electrophysiological studies on errorless and errorful learning [25,26,59] that verbal memory retrieval should be modulated by the stimulation of the prefrontal cortex, and more specifically the left PFC. In case the left PFC is solely involved in memory processes, we expected a better learning performance following anodal stimulation of the left PFC [31,33,34], and we assumed a decrease in performance during cathodal stimulation [46] independent of the learning mode. However, in case the left PFC plays an important role in executive control during memory processes we expected a different modulation following tDCS driven by learning mode as errorful learning compared to errorless learning enhances conflict processing during retrieval as added errors during learning are likely to cause interference at retrieval.

## Results

**Figure 1:**
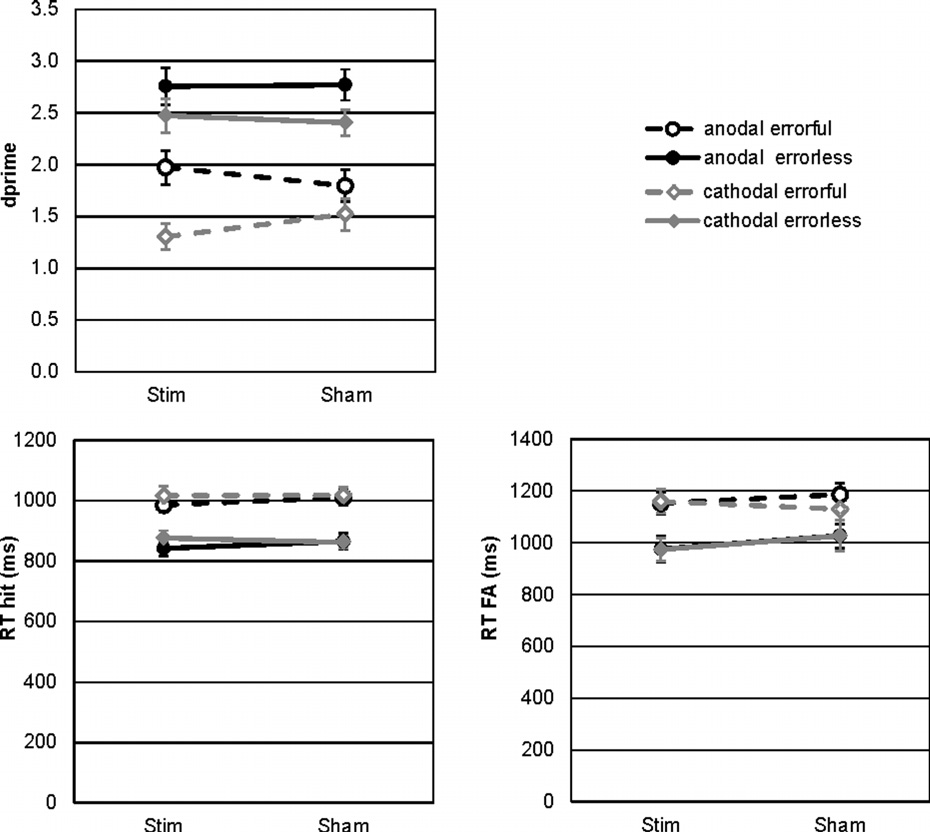
**Behavioral data**. The plots show the memory performance and the reaction times for EL (solid line) and EF (dashed line) learning after anodal (black), cathodal (grey) or sham stimulation.

Performance measures are shown in Figure 1. Following anodal real or sham stimulation, the errorless learning led to an improved memory performance as compared to the errorful learning mode. Errorless learning was not modulated by anodal or cathodal stimulation as real and sham stimulation resulted in similar discrimination indices. Differences can be observed for errorful learning. Following real cathodal stimulation compared to sham, memory performance after errorful learning was reduced (errorful: dprime stimulation < sham) and vice versa for the anodal stimulation (errorful: dprime stimulation > sham).

The ANOVA crossing the between factor Group (anodal vs. cathodal) and the within factors Stimulation (active vs. sham) and Learning Mode (errorful vs. errorless learning) on the dprime measures revealed a group effect (Group: F_1,34 _= 4.59, p < .04), a significant main effect for learning mode (learning mode: F_1,34 _= 136.61, p < .001) and a significant triple interaction (Group × Stimulation × Learning Mode: F_1,34 _= 4.68, p < .04). The other main effects or interactions failed to reach significance (Stimulation: F_1,34 _= 0.04, p > .9; Stimulation × Group: F_1,34 _= 2.35, p > 0.1, Learning Mode × Group: F_1,34 _= 0.81, p > 0.3, Stimulation × Learning Mode: F_1,34 _= 0.17, p > .6).

The learning mode effect was statistically significant for all paired comparisons of errorless > errorful (anodal stimulation: t_17 _= 4.17, p < .002; anodal sham: t_17 _= 6.25, p < .001; cathodal stimulation: t_17 _= 14.23, p < .001; cathodal sham: t_17 _= 8.35, p < .001). The anodal stimulation effect directly comparing stimulation > sham failed to reach significance for both learning modes (errorful: t_17 _= 1.59, p > .1; errorless: t_17 _= -0.13, p > .9). The cathodal stimulation effect with stimulation < sham was significant for errorful learning (t_17 _= 2.35, p < .04) but failed to reach significance for errorless learning (t_17 _= -0.61, p > .5). As equality between the baseline conditions was given (see method section) we directly compared both groups with independent t-tests. Differences were solely found for the errorful learning mode (cathodal < anodal: t_34 _= 3.28, p < .003) but not errorless learning mode (cathodal > anodal: t_34 _= -1.16, p > .3).

The mean reaction times for hits and false alarms are shown in Figure 1. The repeated measures ANOVA crossing the between factor Group (anodal vs. cathodal) and the within factors Stimulation (active vs. sham) and Learning Mode (errorful vs. errorless learning) on the reaction times for hits indicated significantly faster responses for errorless than for errorful items (mean RT errorless: 861 ms, errorful: 1008 ms; learning mode: F_1,34 _= 209.94, p < .001). All other main effects or interactions failed to reach significance (Hits: Group F_1,34 _= 0.28, p > .5; Stimulation F_1,34 _= 0.80, p > .3; Stimulation × Group F_1,34 _= 2.18, p > .1; LM × Goup F_1,34 _= 0.03, p > .8; Stimulation × LM F_1,34 _= 0.26, p > .6 and Stimulation × LM × Group F_1,34 _= 0.13, p > .7). The same pattern was observed for false alarms (mean RT errorless: 1001 ms, errorful: 1156 ms; learning mode: F_1,34 _= 38.86, p < .001) and no further significant effect (Group F_1,34 _= 0.28, p > .6; Stimulation F_1,34 _= 0.80, p > .3; Stimulation × Group F_1,34 _= 2.18, p > .1; LM × Goup F_1,34 _= 0.03, p > .8; Stimulation × LM F_1,34 _= 0.26, p > .6 and Stimulation × LM × Group F_1,34 _= 0.13, p > .7).

## Discussion

The aim of the study was to clarify the role of the left PFC in executive control processes during memory retrieval by comparing two different learning modes (errorless and errorful learning) that tax executive processing to a different degree and anodal and cathodal tDCS to modulate the excitability of the left PFC. Memory processing was driven by learning mode as errorless learning was advantageous over errorful learning. More importantly, cathodal stimulation hampered encoding and memory retrieval after errorful learning but not errorless learning, whereas anodal stimulation did not alter encoding and memory retrieval after errorful or errorless learning.

Errorless learning compared to errorful learning had a profound effect on the memory performance. Additionally, the responses to errorless learned items were faster than to errorful items. These findings underscore the benefits of errorless learning as shown before [24,25,59,71,72]. Following Baddeley and Wilson [24], errorful learning results in increased memory errors due to the enhanced activation of the previously incorrectly guessed items. These will lead to an enhanced conflict during recognition as the activation of the target word and the incorrect guessed words compete with each other. By contrast, errorless learning - as a rather managed learning mode with the aim to avoid errors during learning - has the advantage of less possible distractors that might interfere in later retrieval and thus there is reduced conflict during retrieval. For the given design, one could argue that the active task to generate a sentence with the errorless learned word might be the sole explanation of the errorless learning advantage. However, Rodriguez-Fornells et al. [26] and Heldmann et al. [25] argued that the errorful learning required an additional activity by the participant (i.e. the generation of the candidate words), whereas errorless learning did not, which leads to a different level of processing. In order to equate the level of processing as close as possible we followed the proposal of Heldmann and colleagues [25] to introduce the additional task of sentence generation.

There was no effect of tDCS of the left PFC on encoding and memory performance following errorless learning. Encoding and memory performance after errorful learning was reduced by cathodal as compared to sham stimulation, whereas anodal stimulation did not alter encoding and memory performance after errorful learning.

Anodal stimulation failed to improve memory retrieval irrespective of learning mode in the present investigation, which is different from earlier studies. This might be related to diverse stimulation methods (e.g. position of the reference electrode, stimulation site, type of current, current intensity and duration of stimulation) as pointed out in a thorough review [38]. Additionally, most of the reported positive memory effects after anodal stimulation concern working memory processes [31,34,44,73] and not verbal memory processing. Elmer and colleagues [46] used an auditory word list learning paradigm and did not find a modification of short term learning after unilateral anodal stimulation of the DLPFC compared to sham, which is in accordance with the present finding. Moreover, after cathodal stimulation of the DLPFC Elmer et al. [46] found a decrease of short term verbal learning, which again is in accordance with the decrease of memory performance after errorful learning in the present study. The conforming results might be based on the similarities of both studies: the participants had to learn auditorily presented words and had to recall these later on while the left DLPFC was modulated with anodal or cathodal stimulation. However, both studies differed as well. First of all, we did not stimulate the right DLPFC and we used different stimulation settings (Elmer et al. used an enlarged reference electrode and stimulated with a slightly increased direct current of 1.5 mA instead of 1 mA and a shorter duration with 5 minutes compared to 30 minutes). Additionally, the paradigm was different. While Elmer's participants listened to and repeated the word list three times, our participants either heard the first three letters and the words once and produced a sentence with the word (errorless learning) or heard the first three letters and had to guess the words once, which introduced errors during the learning (errorful learning).

Why did we not find a decrease of memory performance for the errorless condition? As pointed out above the retrieval after errorless learning is supposed to be rather conflict free as errors are avoided during learning and thus, nearly no conflicting memory traces are present in later recognition. This greatly reduces the processing demands for the PFC and thus no effect of stimulation can be seen. By contrast, errorful learning challenges the executive control system as erroneous items from the learning phase compete with the correct items during recognition. We hypothesized that cathodal stimulation reduced executive control of memory resulting in a reduced memory performance. These results dovetail nicely with the Activation-Monitoring framework [74] developed to explain false memories. This framework emphasizes that during retrieval one has to differentiate between highly activated but non-presented critical words and studied words to avoid false memories. By this account false memories, for example in the Deese-Roediger-McDermott paradigm, are due to a failure in monitoring processes differentiating falsely from correctly activated words. In a similar vein, greater activations of the dorsolateral prefrontal cortex during false than true recognition have been interpreted as reflecting monitoring-processes induced by the strong sense of familiarity associated with false memories [e.g. 75]. Differentially spoken, disrupting the processing of the left DLPFC via cathodal tDCS disturbs monitoring processes during memory retrieval and hampers the differentiation between correctly and erroneously words learned during errorful learning.

## Limitations

We would like to mention methodological limitations of the present tDCS protocol. The reference electrode placed in the contralateral supraorbital region (either the anode or the cathode) with the same size of the active electrode can also induce reference specific effects (anodal/cathodal) in parallel to the cathodal/anodal effects of the active electrode. However, the present settings were used in well-established tDCS protocols to modulate the left DLPFC [31,34,76,77]. Another limitation in terms of the stimulation protocol is that the four learning phases and four recognition phases were done in an alternating fashion. We therefore cannot differentiate between the stimulation influences on encoding or retrieval. However, the given protocol appeared to offer the best trade-off between a sufficient number of stimuli and a viable task for participants.

## Conclusions

Altogether we conclude that cathodal tDCS hampered executive control mechanisms during encoding and verbal memory processes and led to effects when retrieval conflict was induced by the errorful learning mode. This underscores the role of the left PFC in the control of encoding and verbal memory retrieval. On a more general level, the present study attests to the potential of the tDCS to study the neural substrates of cognitive functions.

## Methods

### Participants

Forty healthy participants were split into two groups. Four datasets had to be removed due to technical problems with the button responses. The first group (N = 18; age 23.3 +/- 3.0 years; 13 women) received both anodal and sham tDCS over the left prefrontal cortex. The second group (N = 18; age 23.0 +/- 3.4 years; 13 women) received both cathodal and sham tDCS over the left prefrontal cortex. All participants were right-handed, and had no history of neuropsychiatric or cardiovascular disease. Written informed consent was obtained from all participants before they entered the study, and the study protocol was approved by the local ethics committee of Magdeburg.

### Experimental protocol

This study was designed as a single-blind, crossover, sham controlled experiment. All participants participated in both active and sham tDCS. The order of stimulation was counterbalanced and randomized across all participants. To minimize carryover effects, the interval between sessions was seven days minimum. Initially, the participants were familiarized with the cognitive tasks.

### Cognitive paradigm

To evaluate changes in the cognitive control system of memory performance after tDCS, we used a recognition memory task requiring a yes/no response to items that had been acquired during errorless and errorful learning [nearly identical to 25, 26]. Subjects participated in one errorful learning block and one errorless learning block per session (i.e. stimulation or sham session). The order of learning blocks was counterbalanced across subjects. One block comprised two runs each composed of a learning phase and a subsequent recognition phase resulting in two errorless and two errorful runs per session. In each run each participant performed a word-fragment-completion task for 30 word-fragments [identical to 25, 26]. In the errorful condition, the first three letters of a word were given by the experimenter and the subject was asked to guess words to complete this fragment. After guessing, the experimenter revealed which word was the target word to be remembered. If subjects failed to guess the intended target word, the experimenter introduced example words and the target word. For each of the presented word-fragments at least two German words exist with a high and comparable guessing probability, e.g. ANZ: 'Anzeige' [advertisement], 'Anzahl' [number] [25,26]. The assignment of the target candidate for each word-fragment was balanced across subjects. There were four different assignments (see Table 1) which were systematically rotated over participants.

**Table 1:** Example for an assignment of candidate words to the learning lists

Condition	List A	List B	List C	List D
Errorless target	Hafen	Hafer	Anzeige	Anzahl
Errorless non-target	Hafer	Hafen	Anzahl	Anzeige
Errorful target	Anzeige	Anzahl	Hafen	Hafer
Errorful nontarget	Anzahl	Anzeige	Hafer	Hafen
New word	Feder	Feder	Feder	Feder

Translations: Hafen - habor, Hafer - oat, Anzeige - advertisement, Anzahl-number, Feder - feather.

For each fragment one word was used during the learning phase as a target word, while the other high frequency alternative was used as distracter during the recognition phase. In the errorless learning condition the first three letters of the word were introduced by the experimenter directly followed by the target word. During errorful learning the participants guessed several words to complete the word-fragment which resulted in deeper processing of words as compared to errorless word list learning. To ensure such a deeper processing of words in the errorless condition as well, participants had to produce a sentence with the word. During each recognition phase 30 targets, 30 distracters and 30 additional new words were presented in a randomized order. There were two errorless and two errorful runs per session (active or sham tDC-stimulation) resulting in 120 words per stimulus type per session.

The task was to indicate by button press (right index/middle finger), whether or not a given word was a target word. The participants did not receive feedback about the correctness of the actual response. The words were presented in white letters on a black background in the middle of a computer screen. Stimuli subtended 0.57° in height and between 1.7° and 4.9° in width. The stimulus duration was 300 ms with a stimulus-onset-asynchrony between 1800 and 2500 ms. Altogether, each run lasted 15 minutes including the learning and recognition phase.

### Transcranial direct current stimulation application

Direct current was transferred by a saline-soaked pair of surface sponge electrodes (35 cm^2^) and delivered by a specially developed, battery-driven, constant current stimulator (*eldith*, neuroConn GmbH, Ilmenau, Germany). For anodal stimulation of the left dorsolateral prefrontal cortex, the anode was placed over position F3 (according to the 10-20 international system for electroencephalogram electrode placement), and the cathode was placed over the contralateral right supraorbital area [31,34,76,77]. For cathodal stimulation of the left dorsolateral prefrontal cortex, the cathode was placed over F3 and the anode over the contralateral right supraorbital area. A constant current of 1 mA was applied for 30 min starting 10 minutes before the first learning phase. This timing was motivated by the results of Ohn and colleagues [34] who reported an increase of WM accuracy after 20 minutes of anodal stimulation compared to baseline and increased accuracy after 30 minutes of anodal stimulation compared to baseline and sham stimulation with the same stimulation settings (35 cm^2 ^electrodes, 1 mA, anode was placed at F3 position, reference placed at the contralateral right supraorbital area). These effects diminished after an hour of anodal stimulation. Here, the first recognition phase started around 20 minutes after stimulation onset (10 minutes waiting and around 10 minutes learning phase) and the fourth and last recognition phase started at around 65 minutes after stimulation onset. With the present timing of stimulation we sought to tap into the maximal tDCS effects similar to Ohn et al. [34]. After the stimulator had been turned off, the electrodes were kept in place until the end of measurement. For sham stimulation, the same electrode placements were used, but the current was applied for 8 s and was then turned off. This procedure ensured the blinding of the subjects for real or sham stimulation as they also felt the initial itching sensation for the first seconds of tDCS [31,38].

### Data analysis

The primary outcomes of this study were the response times (hits and false alarms) and signal detection measure d' (accuracy). Here we focus on the discrimination index (d') as it describes the subject's ability to discriminate between old and new items in memory recognition [78]. Values for d' were estimated by the z-score of the false alarm rate minus the z-score of the hit rate.

T-tests between the baseline conditions (i.e. sham stimulation) of both groups were calculated in order to evaluate the equality of both groups in baseline performance: The comparisons of the performances of both groups following sham stimulation did not reveal any significant differences in baseline performances between the two groups (errorless: t_34_= -1.38, p > .1; errorful: t_34_= -1.26, p > .2). The reaction times for hits (errorless: t_34_= 0.83, p > .9; errorful: t_34_= -0.19, p > .8) and for False Alarms (errorless: t_34_= 0.02, p > .9; errorful: t_34_= 0.90, p > .3) following sham stimulations were not significantly different between both groups.

Measures were subjected into a repeated measures ANOVA including the between factor Group (anodal vs. cathodal) and the within factors Stimulation (active vs. sham) and Learning Mode (errorful vs. errorless learning). Differences were further analyzed by paired t-tests within groups and by independent t-tests between groups.

## Authors' contributions

AH conceived and designed the experiments, performed the statistical analysis and drafted the manuscript. BM contributed to the design and revised it critically for important intellectual content. MS performed the experiments, contributed to the statistical analysis and revised the manuscript critically for important intellectual content. SS performed the experiments, contributed to the statistical analysis and revised the manuscript critically for important intellectual content. TFM conceived and designed the experiment and drafted the manuscript. All authors read and approved the final manuscript.
